# FMRI-based prediction of naltrexone response in alcohol use disorder: a replication study

**DOI:** 10.1007/s00406-021-01259-7

**Published:** 2021-04-21

**Authors:** Patrick Bach, Georg Weil, Enrico Pompili, Sabine Hoffmann, Derik Hermann, Sabine Vollstädt-Klein, Falk Kiefer, Karl Mann, Wolfgang H. Sommer

**Affiliations:** 1 Department of Addictive Behavior and Addiction Medicine, Medical Faculty Mannheim, Central Institute of Mental Health, University of Heidelberg, Square J5, 68159 Mannheim, Germany; 2 Feuerlein Center On Translational Addiction Medicine (FCTS), University of Heidelberg, Heidelberg, Germany; 3 Institute of Psychopharmacology, Central Institute of Mental Health, Medical Faculty Mannheim, University of Heidelberg, Heidelberg, Germany; 4 Bethanien Hospital for Psychiatry, Greifswald, Germany

**Keywords:** Naltrexone, Cue-reactivity, Alcohol addiction, Relapse, FMRI, Precision medicine

## Abstract

Pharmacological treatment in alcohol use disorder suffers from modest effect sizes. Efforts have been undertaken to identify patient characteristics that help to select individuals that benefit from pharmacological treatment. Previous studies indicated that neural alcohol cue-reactivity (CR) might provide a marker that identifies patients, which benefit from naltrexone treatment.

We investigated the reproducibility of the association between ventral striatum (VS) activation and naltrexone (NTX) treatment response by analyzing data from a recent longitudinal clinical trial in *N* = 44 abstinent treatment-seeking alcohol-dependent patients. A follow-up was conducted over 3 months. We computed the percentage of significant voxels in VS and tested main effects and interactions with NTX treatment on relapse risk using Cox Regression models.

We found a significant interaction effect between pre-treatment cue reactivity in the VS and NTX treatment on time to first heavy relapse (Hazard Ratio = 7.406, 95% CI 1.17–46.56, *p* = 0.033), such that the patient group with high VS activation (defined by a mean split) showed a significant medication effect (Hazard Ratio = 0.140, 95% CI 0.02–0.75, *p* = 0.022) with a number needed to treat of 3.4 [95% CI 2.413.5], while there was no significant effect in the group with low VS activation (Hazard Ratio = 0.726, *p* = 0.454).

Thus, using an independent sample we replicated the previously described positive association between VS activation and NTX efficacy. Although our results should be considered cautiously in light of the small sample size, our results support the potential of neural alcohol CR as a tool for precision medicine approaches in alcohol dependence.

## Introduction

Alcohol use disorder (AUD) is one of the most devastating diseases world-wide and a major risk factor for death, disease and disability [42]. Currently, only a few medications are available for treating patients with AUD. The opioid antagonist naltrexone (NTX) is approved by the Food and Drug Administration (FDA) and the European Medical Agency (EMA) for relapse prevention in AUD. However, treatment with NTX suffers from modest effect sizes with estimated numbers of patients needed to treat (NNT) ranging above 10 for preventing one relapse [25]. Understanding the neural and behavioral mechanisms underlying the highly variable treatment response to anti-relapse medications will be a key factor for improving individual treatment success and enhancing impact on clinical practice [21]. Conceptually, NTX is thought to attenuate a hyperreactivity of the mesolimbic reward system via its actions on the mu-opioid-receptor. In this context, it was demonstrated that hyperreactivity of the mesolimbic reward system can be measured using fMRI cue-reactivity paradigms [9, 12] and association between striatal dopamine receptor availability as well as dopamine activity and fMRI response could be established [24], These findings suggest that high cue-induced brain activation in the mesolimbic system might identify patients with a beneficial response to NTX treatment. Indeed, human brain imaging studies identified drug cue-induced brain response as potential predictor for the response to naltrexone treatment [11]. The German PREDICT study was one of the first large-scale studies that intended to determine neural and behavioral predictors for identifying AUD patients that respond to pharmacological treatment [33]. Results of the study indicated that naltrexone-treated patients with high cue-induced baseline activation in the ventral striatum (VS) showed longer relapse-free survival [36]. Further imaging studies found significant interactions between naltrexone treatment and a reduction of mesolimbic cue-reactivity (CR) in the VS [49, 50], the medial prefrontal cortex (mPFC), the orbitofrontal cortex (OFC), the anterior cingulate cortex (ACC) and the inferior frontal gyrus (IFG) [32]. In addition, patients that showed a reduction in VS cue-reactivity and received naltrexone showed fewer heavy drinking days (%HDD) during follow-up, compared to those receiving placebo [50]. Further studies showed that VS activation predicted alcohol consumption in a subsequent alcohol self-administration paradigm, even after controlling for medication, alcohol use severity and OPRM1 (mu-opioid receptor) genotype [30]. In a recent open-label clinical trial, which compared the effects of NTX against standard treatment, our own research showed that mesolimbic CR significantly increased over 2 weeks of abstinence in the standard treatment group, but not in patients receiving NTX [6]. NTX blocked increases in left putamen CR and reduced relapse risk during the 90-day follow-up.

To date, the analytic approaches across different trials showed substantial variability. Following the principles of replication and validation of scientific findings in independent datasets, we conducted a secondary analysis of our trial data [6] by emulating the analytic approach of the PREDICT study by Mann et al. [36]. Specifically, we set out to test the reproducibility of the interaction between baseline alcohol cue-induced CR in the VS (measured by computing the percentage of active voxels [%AV] in the VS, reflecting the extent of significant activation in this area) and NTX treatment on the risk to relapse to heavy drinking. We hypothesized that higher activation in the VS significantly interacts with NTX, such that patients with high baseline CR show a larger medication effect on risk to relapse.

## Methods and materials

### Study design and patient sample

**Fig. 1:**
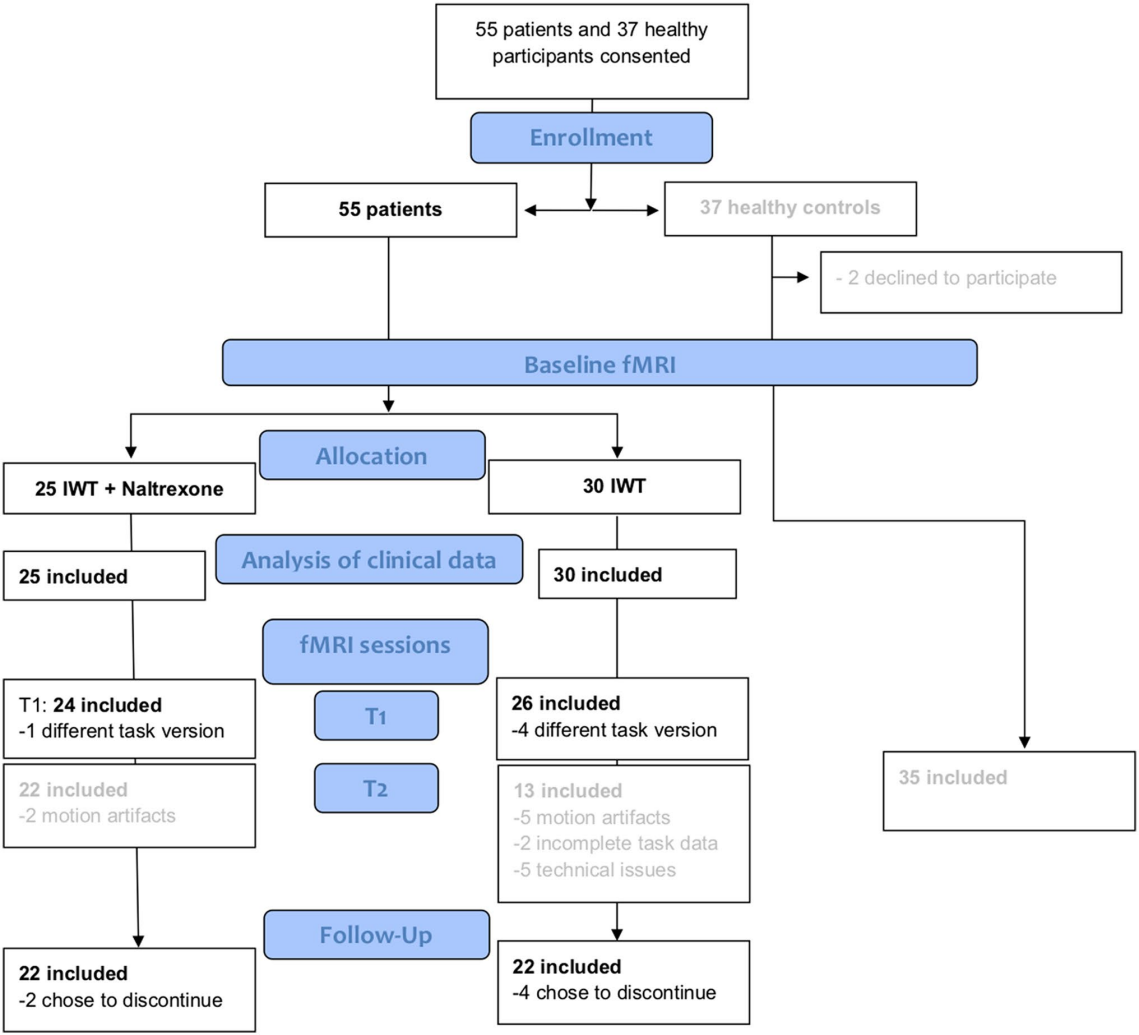
CONSORT diagram of subject flow through the study. Datasets of *n* = 44 patients (*n* = 22 of whom later received Naltrexone [NTX] in addition to intensified withdrawal treatment [IWT]) with complete baseline imaging data and complete follow-up data were included in current analyses. Datasets of other patients with incomplete baseline fMRI data or incomplete follow-up data and datasets of the healthy control sample were not included in current analyses. These data are reported in Bach et al. [6]

We conducted secondary analyses of data, which was collected in the framework of a longitudinal naturalistic clinical trial at the Central Institute for Mental Health (CIMH) in Mannheim, Germany [6]. Current analyses were not formally pre-registered, but followed the principles of replicability of a previous comparable trial [36]. Our trial was pre-registered and designed to investigate neuronal networks in alcoholism, which included analyses of structural and functional connectivity, as well as alcohol cue-induced brain responses (clinical trials id DRKS00003357). Methodological details of the presented alcohol cue-reactivity fMRI study are reported following the recommendations of a recently published ENIGMA consensus paper [13]. Overall, a total of *n* = 55 abstinent treatment-seeking male patients (100% male) with alcohol dependence were enrolled of whom *n* = 45 patients with complete follow-up and fMRI baseline data were included in current analyses (*n* = 5 patients had to be excluded due to fMRI artifacts or due to a different fMRI task version and *n* = 5 additional patients had to be excluded due to loss to follow-up, because of refusal to continue study procedures)(see Fig. 1 for CONSORT flow chart). Inclusion criteria for the patient sample were the following: (i) diagnosis of an alcohol dependence according to the Diagnostic Statistical Manual of Mental Disorders (DSM-IV), (ii) age between 18 and 65 years, (iii) abstinence from any drugs (controlled by urine drug screening), and (iv) an average minimum consumption of at least six drinks per day (i.e. 84 g alcohol, 1 standard drink = 14 g) in the last 90 days before admission. Exclusion criteria were the following: (i) presence of a comorbid axis-I disorders (other than nicotine dependence) during the past 12 months [The Structured Clinical Interview (SCID-I) for DSM-IV [54] was administered to all participants by trained clinical psychologist and/or psychiatrists, in order to rule out any psychiatric comorbidities], (ii) treatment with psychotropic or anticonvulsive medications, (iii) severe neurological or medical conditions (i.e. liver cirrhosis), (iv) ineligibility for MRI scanning (e.g. metal implants), (v) history of severe head trauma, or (vi) changes in vasoactive or antihypertensive medication during the past 7 days. Inclusion criteria for the healthy control participants were: (i) age between 18 and 65 years, (ii) no axis-I disorder except for nicotine dependence [The Structured Clinical Interview (SCID-I) for DSM-IV [54] was administered to all participants, in order to rule out any psychiatric comorbidities], (iii) average alcohol consumption below one drink per day (14 g) and (iv) absence of any exclusion criteria (see above). All participants were dominantly right-handed as assessed using the Edinburgh Handedness Inventory [41].

The trial consisted of a baseline assessment before treatment with naltrexone was initiated and a second assessment after 2 weeks of treatment and a 3-month follow-up. The baseline assessment was scheduled after about 3 weeks of controlled abstinence from alcohol (i.e. abstinence was controlled by daily unheralded breath alcohol tests and also by intermittent analyses of urine ethylglucuronid levels). It consisted of clinical interviews, psychometric assessments and assessment of neural aclohol cue-reactivity using functional magnetic resonance imaging (fMRI) [52]. At baseline, alcohol use during the past 90 days was assessed using the Form-90 structured assessment interview [38]. No biological markers for alcohol use severity were collected. All patients committed to an abstinence-based treatment and could choose to either receive standard treatment only or to receive additionally naltrexone (oral, 50 mg daily) in an open-label free-choice design. None of the patients received additional anti-craving medication (e.g. Acamprosate). The standard treatment consisted of a daily multi-professional medically-supervised therapy schedule here termed Intensive Withdrawal Treatment (IWT) [31]. The second assessment mirrored the baseline assessment and was scheduled after an average of 15.5 (SD = 3.5) days of study treatment. Adherence to medication was ensured by daily supervised intake of medication. After discharge, adherence was monitored during the follow-up by patient reports. All procedures were approved by the local ethics committee and all patients provided informed written consent.

During the 3-month follow-up, we collected relapse data using the Form 90 [38]. Patients on NTX received the medication throughout the follow-up period. We defined heavy relapse as return to heavy drinking of > 60 g alcohol per day for men. In accordance with our earlier studies, we used time to relapse to heavy drinking as outcome variable in our survival analyses [5, 6, 26, 28].

### Assessment of neural alcohol cue-reactivity using fMRI

We used a visual block-design fMRI alcohol cue-reactivity task. All participants were naïve for the alcohol cue-exposure task (i.e. no prior exposure or training) and instructed to pay close attention to the presented images. All participants were allowed to smoke ad-libitum until the fMRI session and no restrictions were applied to food intake or caffeine consumption. Alertness of participants was supervised by study personal throughout the fMRI session. The pictures were used and validated (i.e. higher subjective craving ratings and higher cue-induced brain responses in mesolimbic brain areas during the presentation of alcohol pictures compared to neutral pictures) in previous studies [52] (pictures cannot be shared, due to copyright restrictions). The task consisted of 12 blocks featuring series of five neutral pictures each and 12 blocks featuring series of five alcohol pictures each. Pictures were presented successively for 4 s each. The blocks were presented in pseudo-randomized order. The total task duration amounted to 12–15 min. Visual stimuli were presented using the Presentation® software (Version 16.0, Neurobehavioral Systems Inc., Albany, CA, USA) and MRI-compatible goggles (MRI Audio/Video Systems, Resonance Technology Inc., Los Angeles, CA, USA). No subjective or physiological data on alcohol craving were collected during the fMRI session.

### fMRI acquisition and preprocessing

Functional neuroimaging was conducted using a 3 T whole-body tomograph (MAGNETOM Trio, Siemens, Germany). For each subject, we acquired a total of 303 T2*-weighted echo-planar images (EPI) in transversal orientation of 30° clockwise to the AC-PC line covering the entire brain (TR = 2.41 s, TE = 25 ms, flip angle = 80°, 42 slices, slice thickness = 2 mm, 1 mm gap, voxel dimensions 3 × 3 × 3 mm^3^, FOV = 192 × 192 mm^2^, 64 × 64 in-plane resolution).

All imaging data were processed and analyzed using SPM8 (preprocessing and individual statistics) and SPM12 (second-level group analyses; Wellcome Department of Cognitive Neurology, London, UK). In order to avoid artifacts due to magnetic saturation, the first 5 scans were excluded from the analyses. The remaining 298 scans were corrected for residual geometric distortion on the basis of the acquired magnetic field map, spatially realigned, normalized to a standardized EPI template from MNI (Montreal Neurological Institute, Quebec, Canada), and smoothed using an isotropic Gaussian kernel for group analyses (full width at half maximum: 8 mm). In addition to distortion correction prior to normalization that included affine transformation followed by a nonlinear registration of imaging data to the EPI template, rigid quality checks were implemented for every participant. Data were excluded if there was excessive motion (> 3 degrees rotation or > 3 mm movement in any axis) or if visual inspection indicated poor fitting to the standard EPI template. Within the final dataset, movement parameters did not differ between alcohol and neutral blocks.

### fMRI data analysis

**Fig. 2:**
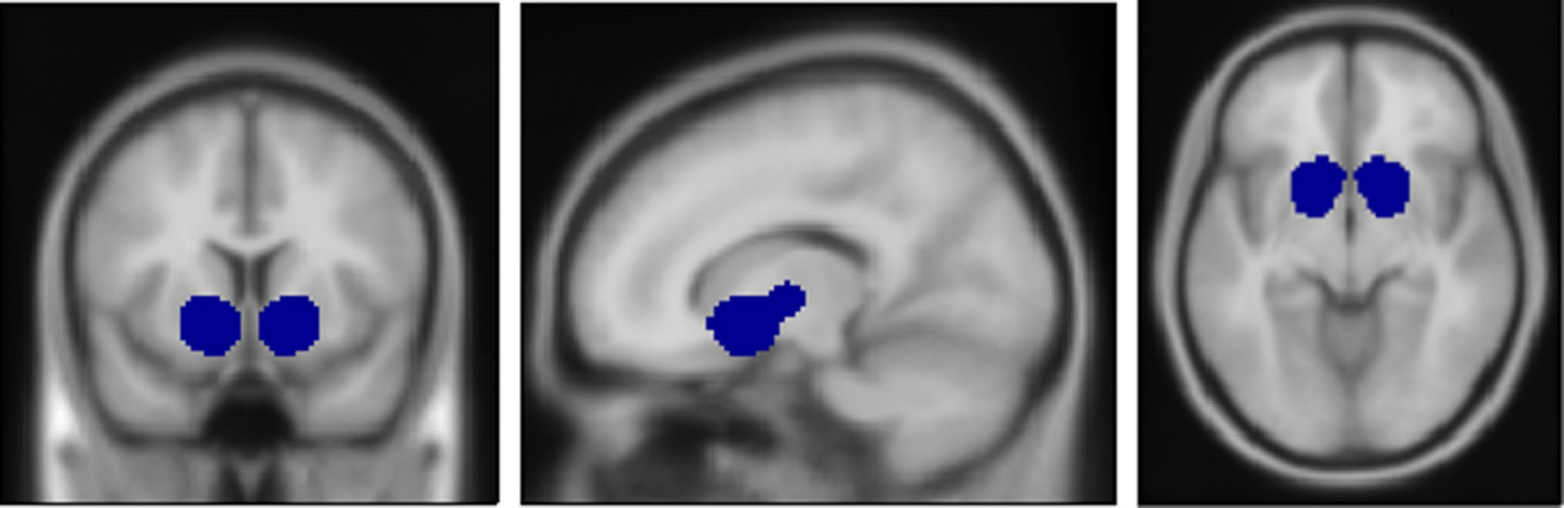
Depiction of the anatomical Ventral Striatum region of interest mask from the Nielsen and Hansen database [39] that was used by Mann et al. [36], which was converted to a binary mask with a threshold of ≥ 0.025, resulting in a total mask size of 2054 voxels

First-level contrast images were computed for all participants by modeling the different task conditions (alcohol, neutral) as explanatory variables within a general linear model and including motion variables as covariates of no interest (i.e. a total of six variables were included reflecting translation and rotation in all three axis). In order to align with the analyses by Mann el al. [36], we computed the percentage of active voxels (%AV) in the VS, defined by an anatomical mask, downloaded from the Nielsen and Hansen database [39], which was converted to a binary mask with a threshold of ≥ 0.025 (see Fig. 2), resulting in a mask size of 2054 voxels. We computed the percentage of active voxels (%AV) in the VS (i.e. the quotient resulting from division of the number of voxels that surpass a statistical significance threshold of p < 0.05 for the contrast “alcohol—neutral” at an individual level (i.e. first-level statistics) by the number of total *n* = 2054 voxels in the VS ROI). Specifically, voxels were considered “active” if the activation value of the voxel surpassed a threshold of p < 0.05 for the contrast “alcohol—neutral”, corresponding to a t-value > 1.65. The percentage of active voxels in the VS (%AV) was computed by dividing the sum of active voxels by the total number of *n* = 2054 voxels in the predefined ROI mask of the VS. Hence, a value of 0 indicates that no voxel in the ROI surpassed the threshold of *p* < 0.05 at the first-level statistics for the contrast “alcohol—neutral”. Deactivation (e.g. negative activation values) and small values that did not surpass the predefined threshold were consequently considered as 0%AV. This aggregation method is an established procedure for analyzing fMRI data. We computed %AV values using a custom script that runs under SPM8 in Matlab, which was repeatedly used in previous studies and is described in detail in [47]. The resulting %AV values were exported into the Statistical Package for the Social Sciences (SPSS, IBM Corp., Somers, NY, USA) version 24.0 for further analyses.

### Analyses of relapse data

In a first step, we tested the reproducibility of previous findings by Mann et al. [36]. Specifically, we tested the main effects and interaction of alcohol CR in the VS and medication on risk to relapse to heavy drinking using a Cox regression models in SPSS. This approach mirrored the analyses by Mann et al. [36]. Descriptive analyses of the values indicated *n* = 1 very extreme outlier (> 3xSD), which was removed from all further analyses (leaving *n* = 44 datasets). As inspection of the %AV indicated that about 30% of the %AV values were close to zero (i.e. < 1) and that the proportional hazards assumption did not hold, we dichotomized the patient group with valid follow-up data (*n* = 45) based on the %AV VS variable into groups with high vs. low %AV brain activation. This procedure mirrored the analytic strategy of Mann et al. [36]. We opted for a mean split to separate groups with high and low %AV values, because a median split would have defined a low value of 3%AV as separator between groups. This value however would not reflect a threshold for defining “high” %AV, as this translates to 61 significant voxels within the VS volume of 2054 voxels. The mean-split defined 9.2%AV as boundary, splitting the total group into *n* = 29 patients with low and *n* = 15 patients with high %AV values in the VS. To validate the chosen threshold for dichotomization, we also conducted additional survival analyses with values ranging from 3%AV to 15%AV in 1% steps.

In a second step, we investigated interaction effects between medication and variables that were implicated in predicting NTX treatment response in previous studies, specifically smoking status and lead-in abstinence [16, 50]. The effect of OPRM1 genotype could not be assessed meaningfully, because the number of G allele carriers was very low and in addition unevenly distributed across groups and reward relief phenotypes could not be assessed, due to the fact that the Inventory of Drinking Situations (IDS) used in the Predict study was not included in the current trial.

Group characteristics and clinical variables of the *n* = 44 patients included in present analyses were analyzed by applying t-tests and Fisher exact tests were appropriate using the IBM SPSS 24.0 software with a predefined statistical threshold of *p* < 0.05.

## Results

### Group characteristics

Of the *n* = 55 patients enrolled in the study, data of *n* = 44 patients, which had valid baseline fMRI data and follow-up data, were included in the current analyses (this sample differs from the sample reported in [6], because the analyses reported there focused on the subset of *n* = 35 patients with two fMRI measurements and analyses of NTX main effects were conducted in all *n* = 49 patients with available relapse data, irrespective of fMRI data availability). Of those patients, *n* = 22 opted to receive NTX and *n* = 22 received IWT only. Both patient groups did not show significant differences on any demographical or clinical variables (see Table 1). Similarly, the groups with high %AV vs. low %AV values in the VS (mean split) did not differ on any demographical or clinical variables (see Table 2). In addition, the current sample characteristics are comparable to the sample investigated by Mann et al. [36] with the current sample reporting slightly higher drinks per day (*p* = 0.558).

**Table 1:** Baseline demographic data, alcohol use and severity measures for patient groups that received standard intensified withdrawal treatment (IWT) or IWT plus Naltrexone (NTX)

	IWT (* n* = 22)	IWT + Naltrexone (*n* = 22)	Statistics	Significance
*Demographical variables*				
Age (years)	42.6 (8.6)	47.3 (8.6)	t_(42)_ = 1.737	*p* = 0.090
Sex (%males)	100	100	–	–
BMI (kg/m^2^)	23.5 (4.2)	26.1 (4.0)	t_(42)_ = 1.952	*p* = 0.059
Education (no post secondary educ./apprenticeship only/ attended college/ higher education)^#^	7/9/3/2	3/14/1/3	F = 3.792	*p* = 0.271
Homeless (yes/no)^#^	2/18	0/22	F = 2.255	*p* = 0.221
Marital Status (married/divorced/single)°	3/6/11	5/9/7	F = 1.948	*p* = 0.420
Number of children (0/1/2/3)^+^	9/5/5/2	9/7/6/0	F = 2.101	*p* = 0.638
Native language (German/Others)^+^	18/3	17/5	F = 0.494	*p* = 0.482
*Substance use patterns*				
Duration of alcohol dependence (years)	11.5 (10.8)	14.8 (10.1)	t_(42)_ = 1.033	*p* = 0.308
Ethanol (g/day; mean of last 90 days)	276.3 (128.1)	217.0 (127.5)	t_(42)_ = 1.499	*p* = 0.142
Drinks per day (mean of last 90 days)	18.8 (11.3)	15.6 (20.6)	t_(42)_ = 0.940	*p* = 0.353
Abstinent days (% in last 90 days)	16.4 (23.9)	15.5 (20.6)	t_(42)_ = 0.137	*p* = 0.891
Heavy-drinking days (% in last 90 days)	81.9 (23.5)	76.9 (26.6)	t_(42)_ = 0.644	*p* = 0.523
Abstinence before Baseline (days)	22.0 (6.5)	25.6 (21.0)	t_(42)_ = 0.750	*p* = 0.458
Smoker (yes/no)	18/4	16/6	F = 1.979	*p* = 0.243
*Clinical scales*				
OCDS (sumscore)	17.6 (8.2)	14.2 (5.8)	t_(42)_ = 1.409	0.168
FTND (sumscore)	6.4 (2.1)	5.3 (2.9)	t_(32)_ = 1.242	0.223
ADS (sumscore)	13.2 (5.7)	13.2 (6.8)	t_(42)_ = 0.034	0.973
STAI (trait sumscore)	42.2 (8.9)	38.9 (10.8)	t_(40)_ = 1.048	0.301
BDI (sumscore)	10.5 (8.3)	10.4 (7.7)	t_(42)_ = 0.023	0.982

*ADS* Alcohol dependence scale, *BDI* Beck depression inventory, *BMI* Body mass index, *FTND* Fagerstroem test for nicotine dependence, *IWT* Intensive withdrawal treatment, *OCDS* Obsessive–compulsive drinking scale, STAI = State-trait-anxiety inventory, SD = standard deviation

* = significant differences between *p* < 0.05

^#^Missing data for *n* = 2 patients

^+^Missing data for *n* = 1 patient

°Missing data fpr *n* = 3 patients

**Table 2:** Baseline demographic data, alcohol use and severity measures for patients with high vs. low alcohol cue-induced activation in the ventral striatum

	Low %AV (*n* = 29)	High %AV (*n* = 15)	Statistics	Significance
Medication (NTX vs. IWT)	12/17	10/5		
*Demographical variables*				
Age (years)	44.2 (9.2)	46.6 (8.1)	t_(42)_ = 0.853	*p* = 0.398
Sex (%males)	100	100	–	–
BMI (kg/m^2^)	2$.5 (4.3)	25.3 (4.5)	t_(42)_ = 0.505	*p* = 0.617
Education (no post secondary educ./apprenticeship only/ attended college/ higher education)^#^	5/16/2/4	5/7/2/1	F = 2.154	*p* = 0.590
Homeless (yes/no)^#^	1/27	1/13	F = 0.256	*p* = 0.561
Marital Status (married/divorced/single)°	5/9/14	3/6/4	F = 1.465	*p* = 0.605
Number of children (0/1/2/3)^+^	13/7/7/1	5/5/4/1	F = 1.271	*p* = 0.799
Native language (German/Others)^+^	25/3	10/5	F = 3.223	*p* = 0.104
*Substance use patterns*				
Duration of alcohol dependence (years)	13.8 (11.3)	11.8 (8.5)	t_(42)_ = 0.558	*p* = 0.580
Ethanol (g/day; mean of last 90 days)	210.3 (130.1)	198.5 (143.1)	t_(42)_ = 0.275	*p* = 0.785
Drinks per day (mean of last 90 days)	17.5 (10.8)	16.5 (11.9)	t_(42)_ = 0.275	*p* = 0.785
Abstinent days (% in last 90 days)	16.4 (23.9)	15.5 (20.6)	t_(42)_ = 0.425	*p* = 0.673
Heavy-drinking days (% in last 90 days)	78.9 (26.3)	80.1 (23.0)	t_(42)_ = 0.147	*p* = 0.884
Abstinence before Baseline (days)	21.1 (6.3)	28.9 (24.7)	t_(42)_ = 1.594	*p* = 0.119
Smoker (yes/no)	23/5	11/3	F = 0.075	*p* = 0.999
*Clinical scales*				
OCDS (sumscore)	17.9 (7.8)	14.0 (5.1)	t_(42)_ = 1.701	*p* = 0.097
FTND (sumscore)	6.5 (2.2)	4.6 (2.8)	t_(32)_ = 1.895	*p* = 0.076
ADS (sumscore)	13.5 (6.9)	12.8 (4.8)	t_(42)_ = 0.307	*p* = 0.761
STAI (trait sumscore)	42.2 (11.1)	37.3 (6.4)	t_(40)_ = 1.535	*p* = 0.133
BDI (sumscore)	11.4 (8.63)	8.6 (6.0)	t_(42)_ = 1.063	*p* = 0.294

Low %AV = patients with percentage active voxels lower or equal to the group mean value of 9.2%. High %AV = patients with percentage active voxels in the ventral striatum higher than the group mean value of 9.2%

*ADS* Alcohol Dependence Scale; *BDI* Beck Depression Inventory; *FTND* Fagerstroem test for nicotine dependence; *IWT* Intensive withdrawal treatment; *OCDS* Obsessive–compulsive drinking scale; *STAI* State-trait-anxiety inventory; *SD* standard deviation

*Significant differences between *p* < 0.05

^#^Missing data for *n* = 2 patients

^+^Missing data for *n* = 1 patient

°Missing data for *n* = 3 patients

### Imaging outcomes

Results show a mean percentage of active voxels (%AV) in the VS of 9.2 (SD = 13.0, median = 3.4) with a range between 0 and 43, which closely corresponds to the data of Mann et al. [36]. At the time of baseline assessment, the presumptive IWT and IWT + NTX groups did not differ with regards to the %AV values in the VS (M_NTX_ = 12.7, SD_NTX_ = 14.7, M_IWT_ = 6.9, SD_IWT_ = 10.7, t = 1.510, p = 0.138). Thus, there was no selection bias between the groups based on their apparent neural cue-reactivity. Whole-brain analyses of imaging data in the whole sample show that alcohol cues compared to neutral cues (contrast: alcohol-neutral) elicited higher brain activation in a network of frontal, temporal, occipital and mesolimbic brain areas, including the superior, middle and inferior frontal gyri, parts of the temporal and parietal gyri, as well the cerebellum, caudate, pallidum and thalamus (see supplementary Table S1). Additionally, analyses of mean alcohol cue-induced activation (contrast: alcohol—neutral), extracted from the VS ROI, show a significant main effect of stimulus category, such that alcohol cues compared to neutral cues induced higher mean activation in the VS ROI (t_(42)_ = 2.126, *p* = 0.039, see Supplementary Fig. S1).

### Relapse to heavy drinking

**Fig. 3:**
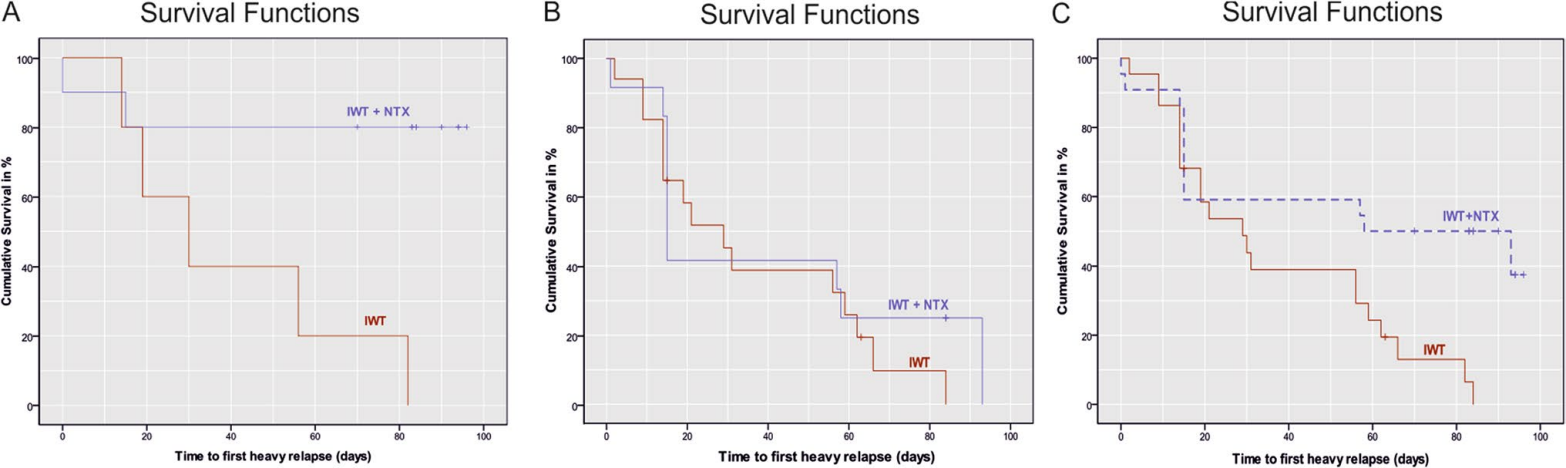
Kaplan Meier curves illustrating: **a** the significant naltrexone effect in patients with high (i.e. > 9.2%) percentage active voxels (%AV) in the ventral striatum (VS) at baseline (*n* = 15, Hazard Ratio = 0.140, 95% CI 0.02–0.75, *p* = 0.022, 7 [47%] patients of this group relapsed and 8 remained abstinent), **b** the absence of a naltrexone effect in patients with low (i.e. < 9.2%) %AV in the VS (*n* = 29, HR = 0.726, 95% CI 0.314–1.679, *p* = 0.454, 25 [86%] patients of this group relapsed and 4 remained abstinent) at baseline, and **c** the longer time to first heavy-relapse in patients receiving naltrexone treatment (*n* = 44, Hazard Ratio = 0.397, 95% CI 0.177–0.809, *p* = 0.012). NTX = Naltrexone, IWT = Intensified Withdrawal Treatment

Of the *n* = 44 patients that were included in current analyses 32 (72%) relapsed to heavy drinking within 90 days after the baseline assessment. While 20 out of 22 patients (90%) on the IWT group relapsed only 12 out of 22 patients (55%) relapsed in the NTX group. Additional analyses showed a significant main effect of %AV in the VS (mean split: high vs. low) on relapse risk (hazard ratio [HR] = 2.957, 95%CI 1.25–6.95, *p* = 0.013), such that the patient group with high %AV (i.e. > mean split) showed a higher relapse risk during follow-up. Additionally, results show a significant interaction between medication and %AV in the VS on relapse risk to heavy drinking during the 90-days follow-up (HR = 7.406, 95% CI 1.17–46.56, *p* = 0.033), such that NTX-treated patients with high CR at baseline, compared to patients with low CR, had a longer time to heavy-relapse. Following, separate survival analyses in the groups with high vs. low %AV in the VS demonstrated a highly significant effect of NTX (vs. IWT only) in the group of patients with high %AV in the VS (*n* = 15, HR = 0.140, 95%CI = 0.02–0.75, *p* = 0.022, 7 [47%] patients of this group relapsed and 8 remained abstinent, see Fig. 3a), whereas no NTX effect was found in the patients with low %AV in the VS (*n* = 29, HR = 0.726, 95% CI 0.314–1.679, *p* = 0.454, 25 [86%] patients of this group relapsed and 4 remained abstinent, see Fig. 3b). Cox regression analyses also demonstrated a significant main effect of medication on risk to relapse to heavy drinking with NTX treatment being associated with a lower risk to relapse (HR = 0.397, 95%CI 0.177–0.809, *p* = 0.012, see Fig. 3c). The HR can be translated to a number needed to treat (NNT) of 3.4 [95%CI 2. 4–13.5] based on the procedure suggested by [1] for the fixed time point of 90 days after study inclusion. Subsequent analyses testing different thresholds between 3%AV and 15%AV for defining high vs. low %AV groups validated the chosen group-defining threshold of 9.2%AV, showing that only group-defining thresholds of 8%AV 9%AV and 9.2%AV resulted in statistically significant Cox regression models (HR_Interaction5%AV_ = 2.820, p = 0.186, *n* = 18 high vs. *n* = 26 low %AV; HR_Interaction6%AV_ = 2.820, *p* = 0.186, *n* = 18 high vs. *n* = 26 low %AV; HR_Interaction7%AV_ = 5.205, *p* = 0.052, *n* = 16 high vs. *n* = 28 low %AV; HR_Interaction8%AV_ = 7.406, *p* = 0.033, *n* = 15 high vs. *n* = 29 low %AV; HR_Interaction9%AV_ = 7.406, *p* = 0.033, *n* = 15 high vs. *n* = 29 low %AV; HR_Interaction10%AV_ = 4.983, *p* = 0.154, *n* = 13 high vs. *n* = 31 low %AV; HR_Interaction11%AV_ = 4.983, *p* = 0.154, *n* = 13 high vs. *n* = 31 low %AV; HR_Interaction12%AV_ = 3.243, *p* = 0.219, *n* = 12 high vs. *n* = 32 low %AV; HR_Interaction13%AV_ = 3.243, *p* = 0.219, *n* = 12 high vs. *n* = 32 low %AV; HR_Interaction14%AV_ = 2.425, *p* = 0.355, *n* = 11 high vs. *n* = 33 low %AV; HR_Interaction15_%AV = 2.425, *p* = 0.355, *n* = 11 high vs. *n* = 33 low %AV; similar statistical values for different %AV thresholds result from similar grouping in high vs. low %AV groups for those values).

Cox regression models testing the interactions between medication and additional variables that were implicated in predicting NTX treatment response, specifically lead-in abstinence (HR = 0.533, 95% CI 0.241–1.179, *p* = 0.120) and smoking status (HR = 0.984, 95% CI 0.955–1.015, *p* = 0.315), did not show a significant interaction effect with NTX on time to first heavy relapse.

### Discussion

Following the idea that identifying the neurobiological and behavioral correlates of dopamine-mediated reward processing could provide insights into markers that identify patients that benefit from NTX treatment [20, 22, 34], we investigated the reproducibility of the associations between extent of neural alcohol-cue reactivity in the VS (specifically the percentage of significantly activated voxels in this regions) and NTX treatment response. Indeed, we could replicate the findings of the previous work by Mann et al. [36] in a well-characterized clinical sample of alcohol-dependent patients using the same analytical strategy. We could confirm that patients with higher baseline %AV in the VS benefited from treatment with NTX. Specifically, we replicated the finding of previous studies that NTX significantly reduced relapse risk in patients with high alcohol cue-induced activation in the VS. In line with previous work, there was no significant NTX effect in patients with low alcohol cue-induced activation in the VS. Our replication study supports the reproducibility of the findings by Mann et al. [36]. Taken together, our data support the notion that alcohol cue-induced VS activation is a potential marker for identifying patients that benefit from NTX treatment. In contrast to other measures of brain activation, the %AV values are defined based on individual first-level imaging brain maps and all voxels in a pre-defined region of interest are considered separately and equally with regard to whether or not they surpass a pre-set statistical threshold. Hence, even though mean activation in a specific ROI might be negative (see also Supplementary Fig. S2), potentially due to outlying values, the %AV can still be positive, because all voxels surpassing the pre-defined threshold are counted. Hence, this measure is less prone to bias by extreme values (see also Supplementary Fig. S3). The %AV value takes into account the spatial extent of significant activation in a certain brain region, while other measures, such as the mean activation value take into account the intensity of activation in a brain area. Previous studies indicated that the %AV value is especially suitable and more robust than other ROI aggregation measures in populations with high inter-subject variation in noise. The analyses by Reinhard et al. [47] demonstrated that the %AV values showed descriptively better predictive capacity with regard to predicting relapse risk, based on alcohol cue-induced activation in the VS, compared to other measures, i.e. mean activation values. Previous reports also pointed towards the problem that mean activation values bear the risk of bias due to outliers. Additionally, in areas where activation and deactivation are observed concurrently, these might cancel each other out, resulting in zero values [45]. Hence, the percentage of active voxels might be more suitable for predicting relapse risk, compared to other ROI aggregation methods.

Compared to other potential clinical and neurobiological markers, the neural alcohol CR might be a particularly suitable marker for investigating NTX effects, due to its association with mesolimbic dopamine activity [24], which is an indirect target of NTX actions, via its effects of the opioid system. The notion that NTX exerts its effects on the dopamine system via its effects at the mu-opioid receptor is supported by animal studies [37, 51]. In humans, the hypothesis of opioid-dependent dopamine release in response to alcohol is supported by the finding that a genetic variation at the mu-opioid receptor gene locus (OPRM1) influences alcohol-induced dopamine release in the VS [46].

Additionally, neural alcohol CR shows close associations with clinical symptoms (e.g. craving) and patient outcome (e.g. relapse risk) [4, 5, 7, 12, 19]. Beyond this, our own study demonstrated that neural alcohol CR in the left putamen responded to NTX treatment, such that NTX blocked the increase in CR in this area over 2 weeks of treatment, which was observed in the IWT group [6]. Importantly, an increase in neural alcohol CR in the left putamen was associated with increased relapse risk, while a decrease or stable activation predicted lower relapse risk. Schacht and colleagues (2017) previously demonstrated that NTX vs. placebo reduced alcohol CR in the VS over 2 weeks of treatment and that patients showing a reduction in CR showed fewer %HHD under NTX treatment compared to those with an increase in neural CR. These observations support the idea that neural alcohol CR might not only serve as surrogate marker for identifying subgroups of patients that benefit from NTX treatment, but might also be sensitive to NTX effects, thus being capable of capturing NTX effects on neural brain activation, which—according to previous work [6, 50]—could be a mechanism via which NTX exerts its relapse preventing effects. This suggests that neural alcohol CR might provide characteristics that are distinct and favorable compared to other potential markers for NTX treatment efficacy. According to this idea, the variable NTX efficacy might in part be due to the observation that only about a half of the patients show a positive alcohol CR in the mesolimbic system [6, 27]. Importantly, the extent of neural alcohol CR does not seem to be related to patient’s choice of NTX vs. standard treatment, suggesting that patients do not “self-select” and other markers are needed, when conducting fMRI is not feasible [6].

In this regard, studies indicated that factors beyond neural CR might predict NTX efficacy in patients. Prominently, but not undisputed, the OPRM1 polymorphism A118G (rs1799971) was associated with treatment effectiveness in several trials [3, 8, 43], while other studies could not confirm the association [10, 18, 44, 50]. Also, in the study by Mann et al. [36], there was no significant effect of the OPRM1 genotype [36]. Beyond that, it was hypothesized that NTX might be most efficacious in patients, who drink alcohol primarily in the context of positive reinforcement and reward [23, 33], while being less effective in patients who drink in the context of negative reinforcement. Previous work, using the large datasets of the COMBINE (Combined pharmacotherapies and behavioral interventions for alcohol dependence) and PREDICT studies, could demonstrate that patient subgroups with primarily reward (i.e. individuals whose drinking is driven primarily by positive reinforcement) or relief drinking (i.e. individuals whose drinking is primarily driven by negative reinforcement) phenotypes could be identified based on the Inventory of Drinking Situations (IDS) [2, 35, 53] and also the Alcohol Abstinence Self-Efficacy Scale (AASE) [48], basically defining four phenotype groups: low reward and relief, high reward and relief, high relief and low reward, and high reward and low relief. A study by Mann et al. could show better NTX response in patients with high reward and low relief phenotypes [35] the association between reward and relief phenotypes with NTX treatment response could be replicated by Witkiewitz et al. [53] using data from a 12-week randomized trial in problem drinkers. In addition to these factors, studies indicated that abstinence prior to naltrexone treatment [17, 40], or contrarily active drinking status [29], or smoking status [16, 50] might predict NTX efficacy. Importantly, many of these factors were also associated with the magnitude of neural fMRI BOLD response in general [14] and alcohol CR in specific (abstinence [15], OPRM1 genotype [5, 49]). It can be speculated that some of the inter-individual variance associated with the above stated factors might also reflect in neural alcohol CR. This might also—at least in part—explain why we could not find significant interactions between the aforementioned factors and medication on relapse risk in the current study, when considering %AV in the VS in our models.

### Limitations

Current results should be considered in the light of the limited sample size. Even though we could demonstrate the hypothesized interaction between alcohol cue-induced brain responses and naltrexone treatment on relapse risk, small to medium effects might have remained undetected. Thus, presented results should be validated in larger samples. Here, we intended to replicate and validate the findings from previous studies investigating the interaction between NTX and alcohol CR in the VS [36], by applying the same analytic approach in a comparable sample. However, we were not able to implement continuous %AV values in our Cox regression models as in the reference study, because necessary assumptions (i.e. proportionality of hazards) were not met. In accordance with the analytic strategy that was applied secondary to testing continuous %AV values in the work by Mann et al. [36], we dichotomized %AV values. Due to the distribution of %AV values, we opted to perform a mean split over a median split, in order to move the value for separating the patient groups from 3% AV (translating to 61 active voxels in the ROI mask) to 9.2% AV (translating to 188 active voxels in the ROI) mask, because we argue that values < 9.2% AV are not compatible with the idea of “high” activation by means of a substantial extent of significant voxels in the ROI. It should be noted that there was no significant whole-brain effect of stimulus category on cue-induced VS activation. However, significant whole-brain effects in the VS are not a stringent necessity for the presented analyses, since the %AV values are derived from individual first-level statistics. Furthermore, analyses of mean activation in the VS ROI indicated s significant main effect of stimulus category in this region, supporting the notion of cue-specific activation in this area. In addition, the robustness and validity of the applied alcohol cue-reactivity paradigm itself has been demonstrated repeatedly in previous studies [5, 26, 27, 52]. It was beyond the scope of the current trial to assess cue-induced alcohol craving. Still, investigating cue-induced craving in future studies could be informative, as reward and relief drinking phenotypes—a construct reflecting different drinking motivations—repeatedly demonstrated significant associations with NTX treatment responses [35, 53], rendering associations with alcohol craving likely. Current data were derived from a naturalistic open-label trial design. While this approach aimed a closely reflected current clinical practice (i.e. NTX as add-on to IWT based on a patient’s informed consent), it comes with several limitations, such as the possibility of a selection bias. However, given that the two patient groups did not differ with regard a number of clinical and psychometric characteristics, we believe that the reported NTX main effect was not affected by selection bias. Still, it should be noted that data on, e.g. income levels and employment status were not available for the current sample, leaving open the possibility that patient groups might have differed on these variables. Additionally, even though there was no indication that choice of NTX was driven by external factors (e.g. court sanctions), there might have been reasons for choosing NTX which could not be specified based on the data of the current trial. In addition, data on continuing care during the follow-up period were not assessed in detail. While none of the patients reported participating in any specific form of addiction treatment (e.g. psychotherapy or in-patient treatment) during follow-up, visits to general doctors and self-help group attendance were not assessed. Future studies are needed, in order to systematically assess the potential impact of the aforementioned variables on treatment outcome and their potential interaction with medication and alcohol cue-induced brain responses. In addition, we could not detect significant main effects or interactions between lead-in abstinence, smoking status and NTX treatment on relapse risk in the current sample, but results should be interpreted in the light of the limited sample size. Randomized controlled trials are needed to confirm current findings.

## Conclusion

We could replicate the association between VS activation and NTX efficacy by demonstrating a significant interaction between baseline alcohol CR in the VS and NTX treatment response that was shown previously [36], such that NTX was more efficacious in patients with high %AV in the VS compared to low %AV. Our findings align with previous studies and support the reproducibility and potential of neural alcohol CR for identifying patients that benefit from NTX treatment. Additionally, neural alcohol CR seems to provide several favorable characteristics (e.g. prediction of and sensitivity to NTX effects) that supports its use in identifying subgroups that benefit from NTX treatment. Further research on the topic of precision medicine is needed to identify the relative contribution of neural alcohol CR and variables that predicted NTX efficacy (e.g. smoking status, reward/relief phenotypes, abstinence, OPRM1 status) in previous trials, in order to confirm the relevance of clinical parameters and psychometric scales, which may help to guide decisions on NTX treatment in clinical practice when fMRI is not feasible.
